# Reirradiation of recurrent brain tumors: a review by the ESTRO CNS Focus Group

**DOI:** 10.1016/j.ctro.2026.101260

**Published:** 2026-08-10

**Authors:** Raffaella De Pietro, Elena Clerici, Maciej Harat, Karthica Indramohan, Tomas Kazda, Slavka Lukacova, Andrada Turcas, Giuseppe Minniti, Maximilian Niyazi, Felix Ehret

**Affiliations:** a The European Organisation for Research and Treatment of Cancer (EORTC) Headquarters, Brussels, Belgium; b Radiotherapy and Radiosurgery Department, IRCCS Humanitas Research Hospital, Milan, Italy; c Department of Radiation Oncology, Franciszek Lukaszczyk Oncology Center and Department of Clinical Medicine, Faculty of Medicine, University of Science and Technology, Bydgoszcz, Poland; d Radiotherapy and Proton Beam Therapy Department, University College London Hospital, London, United Kingdom; e Department of Radiation Oncology, Masaryk Memorial Cancer Institute and Faculty of Medicine, Masaryk University, Brno, Czech Republic; f Department of Oncology, Aarhus University Hospital, Aarhus, Denmark; g Department of Clinical Medicine, Aarhus University, Aarhus, Denmark; h Institute of Advanced Studies in Science and Technology, Babeș-Bolyai University, Cluj-Napoca, Romania; i Radiotherapy Department, Oncology Institute “Prof. Dr. Ion Chiricuta”, Cluj-Napoca, Romania; j Department of Radiological Sciences, Oncology and Anatomical Pathology, Sapienza University of Rome, Rome, Italy, IRCCS Neuromed, Pozzilli (IS), Italy; k Department of Radiation Oncology, University Hospital Tübingen, Tübingen, Germany; l German Cancer Consortium (DKTK), partner site Tübingen, a partnership between DKFZ and University Hospital Tübingen, Germany; m Center for Neuro-Oncology, Comprehensive Cancer Center Tübingen-Stuttgart, University Hospital Tübingen, Tübingen, Germany; n Charité – Universitätsmedizin Berlin, Corporate Member of Freie Universität Berlin and Humboldt-Universität zu Berlin, Department of Radiation Oncology, Berlin, Germany; o German Cancer Consortium (DKTK), partner site Berlin, a partnership between DKFZ and Charité – Universitätsmedizin Berlin, Germany

**Keywords:** Reirradiation, *Re*-irradiation, Radiotherapy, Radiation therapy, Brain tumors, Meningioma, Ependymoma, Brain metastasis, Pituitary neuroendocrine tumors, PitNET

## Abstract

**Purpose:**

Reirradiation has become an area of increasing clinical and scientific interest, particularly in neuro-oncology. This non-systematic review on central nervous system (CNS) tumors by the CNS focus group of the European Society for Radiotherapy and Oncology provides a pragmatic synthesis of the available evidence, excluding gliomas. We address key questions related to patient selection, fractionation, technical considerations, and treatment safety.

**Methods:**

Relevant studies on the use of reirradiation for meningiomas, ependymomas, brain metastases, and pituitary neuroendocrine tumors, as well as on the management of radiation necrosis, have been reviewed and summarized.

**Results:**

Reirradiation has been used with varying success in primary and secondary brain tumors. The appropriate use of reirradiation requires consideration of numerous factors, including, but not limited to, prior treatment courses, fractionation, dose to organs at risk, imaging findings, the volume of the recurrent tumor, and the interval since prior treatments. The vast majority of available studies are limited by their retrospective design, lack of standardization in reporting, and overall heterogeneity across cohorts and treatments.

**Conclusion:**

Several reirradiation studies across different tumor entities suggest potential efficacy and safety in carefully selected patients. However, important limitations remain, underscoring the need for well-designed prospective studies with rigorous reporting in both primary and secondary brain tumors.

## Highlights

• ESTRO CNS Focus Group review on CNS reirradiation excluding gliomas.
• Reirradiation shows potential efficacy and safety in carefully selected patients.
• Optimal patient selection, fractionation, dose, and timing remain key determinants.
• Studies are mostly retrospective and heterogeneous.
• Prospective standardized trials with rigorous reporting are warranted.

## 1 Introduction

Despite advances in multimodal therapy, including surgery, radiation therapy (RT), and systemic treatments such as targeted therapy and immunotherapy, a substantial proportion of patients experience disease relapse [1]. Management of recurrent brain tumors, whether primary or metastatic, remains a challenge in neuro-oncology [1]. In the recurrent setting, therapeutic options are often limited, and no universally accepted standards exist. Therefore, management must be tailored on a case-by-case basis, considering prior treatments, tumor type, the extent, size and location of the recurrent disease, patient performance status, and comorbidities. In this setting, multidisciplinary collaboration is not optional but central to guiding safe and effective shared treatment decision-making [1].

Advances in imaging and radiation delivery techniques have broadened the therapeutic window, particularly concerning the use of reirradiation [2], [3], [4]. Reirradiation is not a uniform clinical entity and may encompass a spectrum of clinical scenarios with different geometric, dosimetric, and biological implications. According to the recent European Society for Radiotherapy and Oncology (ESTRO) - European Organisation for Research and Treatment of Cancer (EORTC) consensus, reirradiation may involve situations with direct geometric overlap between the current and previous irradiation volumes (type 1 reirradiation), as well as cases without overlap but with concerns related to cumulative dose exposure of close organs at risk (type 2 reirradiation) [3]. In the central nervous system (CNS), the clinical relevance of reirradiation depends not only on the prescribed dose and fractionation schedule, but also on the spatial relationship between the recurrent lesion, the previously irradiated target volume, and adjacent critical neural structures. Consequently, in-field recurrences, marginal recurrences, and recurrences arising close to vulnerable organs at risk, such as the optic apparatus or brainstem, may represent substantially different retreatment scenarios. Since the majority of the available literature predates the publication of the ESTRO-EORTC consensus, most studies do not provide a formal classification of reirradiation as type 1 or type 2. Therefore, all studies reporting outcomes of CNS reirradiation were considered herein, irrespective of the reirradiation type, while acknowledging the heterogeneity of the reported clinical scenarios.

Modalities such as intensity-modulated radiation therapy (IMRT), volumetric modulated arc therapy (VMAT), proton therapy, and stereotactic radiation techniques, including stereotactic radiosurgery (SRS) and fractionated stereotactic radiation therapy (FSRT), allow for a conformal treatment delivery while sparing surrounding normal brain tissue, making reirradiation a feasible salvage strategy in selected patients. Particle therapy, in particular proton therapy, offers a distinct dosimetric advantage compared with photon-based techniques due to the physical properties of particles, including the Bragg peak, resulting in reduced exit dose and lower integral dose to the surrounding normal brain tissue. This characteristic may be particularly relevant in the reirradiation setting, where cumulative dose constraints to critical structures are a major limitation. Retrospective institutional series have reported encouraging local control and a generally acceptable short-term toxicity profile in intracranial tumors [5]. However, the available evidence in adult CNS reirradiation remains limited and highly heterogeneous. Given the theoretical reduction in long-term neurocognitive and visual impairment, for instance, proton therapy may be particularly relevant for younger patients, those with high cumulative doses near tolerance thresholds, or lesions near the optic apparatus if a steep dose gradient is required, which is often necessary in the reirradiation of skull base meningiomas, pituitary neuroendocrine tumors (PitNETs), and ependymomas. Therefore, the choice between photon and proton techniques should be individualized and based on dosimetric advantages, availability, cost considerations, and the spatial pattern of prior radiation exposure. While proton therapy may offer potential benefits in reducing low- and intermediate-dose exposure to normal brain tissue, its clinical advantage in the reirradiation setting has not yet been definitively established and requires further prospective validation. Nonetheless, several critical issues and questions in the field remain unresolved: the optimal dose and fractionation regimen, timing of treatment and combination with systemic agents, cumulative dose mapping and constraints, imaging registration, tissue recovery and repair capacity, the risk of acute and late adverse effects, and comprehensive reporting [1], [2], [3], [4].

Normal tissue recovery is a critical topic in CNS reirradiation. Clinical and radiobiological evidence suggests that tolerance depends on both the organ at risk and the time interval between treatments. According to international recommendations and prognostic scores for glioblastoma patients, reirradiation should be considered at least 6 months after the first course of RT [6]. This is broadly reflected in clinical practice. In a recent survey, 53% of participants considered an interval of 6–9 months as the minimum acceptable time when fields overlap, i.e., type 1 reirradiation, whereas 25% regarded 3–6 months as sufficient, and 20% recommended waiting more than 1 year [7]. Regardless of the retreatment interval, the optic nerves and chiasm demonstrate limited recovery potential, and the risk of radiation-induced optic neuropathy (RION) increases steeply when the dose exceeds 55–60 Gy (equivalent dose in 2 Gy per fraction [EQD2], α/β 2 Gy) [8]. Visual side effects and radiation necrosis (RN) remain the most feared complications, although modern conformal techniques like VMAT can help to reduce the dose to organs at risk [9]. In primary stereotactic settings, commonly cited maximum radiation doses associated with a < 1% RION risk are 10 Gy in 1 fraction, 20 Gy in 3 fractions, and 25 Gy in 5 fractions [8], [10]. Fractionated stereotactic regimens (21–25 Gy in 3–5 fractions) are generally favored for tumors near the optic pathways, since prior conventional RT often delivers doses approaching the tolerance level. According to the HyTEC (High Dose per Fraction, Hypofractionated Treatment Effects in the Clinic) analysis, prior irradiation is associated with a markedly higher risk of RION after SRS/FSRT, with a >10-fold increase in crude rates, although the exact magnitude of this increase is uncertain due to limited data and multiple confounding factors [10]. Therefore, careful review of prior RT plans, interpretation of cumulative dose, and individualized assessment of recurrence location relative to critical structures are essential before offering reirradiation.

This non-systematic review of the CNS focus group of the ESTRO provides a pragmatic synthesis of the available evidence, addressing questions related to patient selection, fractionation, technical considerations, and treatment safety. The discussion encompasses meningiomas, ependymomas, brain metastases, PitNETs, and the management of RN, while excluding supratentorial gliomas and glioblastomas, which are discussed elsewhere [11]. The goal is to offer an updated, clinically oriented framework to support informed decision-making in complex reirradiation scenarios across various brain tumors.

## 2 Meningioma

In intracranial meningiomas, RT represents a key component of multimodal management, particularly in higher-risk subgroups such as World Health Organization (WHO) grade 2 and 3 tumors [12], [13]. The management of recurrent intracranial meningiomas after RT remains challenging, and reirradiation has emerged as a therapeutic option when surgery is not feasible, or recurrence occurs in anatomically complex regions like the skull base [14], [15]. A recent systematic review and meta-analysis has suggested that reirradiation may be a safe and effective treatment option in selected patients, although the substantial heterogeneity of the available studies — including treatment techniques, treatment intervals, WHO grades, target volumes, fractionation schemes, and toxicity reporting — limits the ability to clearly identify the patient subgroups that benefit most from this approach [16]. Therefore, further studies, particularly prospective registries and clinical trials, are warranted to better define the efficacy and safety of reirradiation in meningioma.

Recurrent meningiomas exhibit heterogeneous biological behavior and clinical outcomes, with WHO grade, proliferative index, anatomical location, and molecular as well as genetic factors influencing prognosis and radiosensitivity. Reirradiation must be tailored to the recurrence pattern, prior dose distribution (type 1 vs. type 2 reirradiation), and the patient's clinical condition and preferences. Surgical resection remains an option for selected cases but is limited by treatment-related morbidity. Current European Association of Neuro-Oncology (EANO) guidelines recommend SRS or FSRT when recurrent disease is amenable to high-precision techniques that spare critical structures [17].

Selecting the appropriate reirradiation technique requires weighing target size, proximity to organs of interest, and prior treatment characteristics. SRS offers steep dose gradients and is typically reserved for small, well-demarcated lesions, whereas FSRT provides improved safety for larger volumes or lesions abutting critical structures due to the radiobiological advantages of fractionation in exploiting differences in repair capacity between normal brain and tumor cells [18]. Comparative planning studies evaluating proton and photon retreatment consistently show that both modalities can achieve comparable coverage of the recurrent target and similar protection of adjacent organs at risk. Consequently, any potential dosimetric advantage varies substantially across patients, depending on the anatomical configuration of the recurrence, the target volume, and the distribution of the initial dose [19].

A growing body of literature has evaluated reirradiation outcomes, suggesting worse progression-free survival (PFS) in WHO grade 2 and 3 tumors compared with grade 1 lesions (Table 1). Early evidence from Wojcieszynski et al. demonstrated markedly inferior one-year control for high-grade histologies, highlighting the aggressive nature of grade 2 and 3 meningiomas and the limitations of salvage RT in this setting [20]. Similar findings have been reported by Kim et al., who observed significantly reduced PFS in grade 2 and 3 tumors after a second course of radiosurgery, suggesting that dose escalation may be required for higher-grade disease [21]. Lin et al. confirmed these trends in a larger cohort, reporting inferior outcomes in aggressive histologies but also highlighting the importance of performance status as a predictor of survival [22]. Haisraely et al. analyzed a retrospective series of 34 patients with anaplastic meningioma who underwent reirradiation; 12 patients (35.3%) received concomitant bevacizumab. This combination resulted in favorable PFS rates and a reduction in RN [23]. These findings have recently been reinforced by the largest multicenter series to date. In that analysis, FSRT, longer intervals between radiation courses, and a higher biologically effective dose (BED) to the tumor at reirradiation were associated with improved tumor control. In contrast, larger tumor volumes were linked to an increased risk of acute and late adverse events, including RN [14].

**Table 1:** Selected studies on reirradiation of meningiomas.

Author/Year	Study design	N patients	WHO Tumor grade	First Course RT technique(s)	First Course RT dose (Gy)/fractions (fx)	*Re*-RT technique(s)	Re-RT dose (Gy)/fractions (fx)	Outcomes (PFS)	RN
Kim et al., 2017	Retrospective	33	1–3	SRS	12.58 Gy (range 10–17)	SRS	Mean dose 14.21 Gy (10–18 Gy)	PFS 1-yr: 85% 2-yr: 60%	6.1% (2 patients)
Lin et al., 2018	Retrospective	43	1–3	EBRT, SRS, FSRT	Prior RT tumor dose in EQD2 53 Gy (35–88 Gy), α/β: 3.28	EBRT, SRS, FSRT	Median Re-RT tumor dose in EQD2 58 Gy (35–88 Gy), α/β: 3.28	PFS 2-yr: 63%	10% (4 patients, grade 3–4)
El Shafie et al., 2018	Retrospective	42	1–3	EBRT, SRS, FSRT	Median dose of 52.9 Gy (12.1–62.4 Gy) for IMRT, 54 Gy (50.5–55.8 Gy) for 3DCRT, 12.1 Gy (12.0–17.0 Gy) for SRS, 58.8 Gy for FSRT	PT, carbon ion therapy	Median 51 Gy (RBE) (15–60 Gy) with a median of 19 fx (range 5–32 fx)	mPFS: 34.3 mo PFS 1-yr: 71% PFS 2-yr: 56.5%	7.1% (3 patients)
Imber et al., 2019	Retrospective	16	1–3	EBRT, SRS	Estimated median dose of prior RT 54 Gy (13–65.5 Gy)	PT	Median 60 Gy (RBE) (30–66.6 Gy)	mPFS: 22.6 mo PFS 1-yr: 80% PFS 2-yr: 43%	13% (only 1 patient symptomatic)
Desideri et al., 2024	Retrospective	181	1–3	EBRT, SRS, FSRT, PT	Median RT Tumor EQD2 43.1 Gy (range 22.5–142.5), α/β: 4	SRS, FSRT, EBRT, PT	Tumor EQD2: 43.1 Gy (19.5–99.4 Gy) Tumor BED: 64.7 Gy (24–149.1 Gy), α/β: 4	PFS 3-yr: 51.6%	Symptomatic: 6.6% (12 patients)
Haisraely et al., 2024	Retrospective	34	3	EBRT	Median dose of 60 Gy (54–60 Gy)	EBRT	Median dose 48.8 Gy BED, α/β: 10	PFS 1-yr: 61.9%, mPFS: 23 mo (for higher doses) BED >50 Gy associated with improved outcomes	Symptomatic: 20.5%, none with bevacizumab

**BED**, biologically effective dose; **EBRT**, external beam radiation therapy; **EQD2**, equivalent dose in 2-Gy fractions; **FSRT**, fractionated stereotactic radiation therapy; **fx**, fractions; **mo**, months; **mPFS**, median progression-free survival; **N**, number; **PFS**, progression-free survival; **RBE**, relative biological effectiveness; ***Re*-RT**, reirradiation; **RN**, radiation necrosis; **RT**, radiation therapy; **SRS**, stereotactic radiosurgery; **IMRT**, intensity modulated radiotherapy; **3DCRT**, three-dimensional conformal radiation therapy; **PT**, proton therapy; **yr**, year.

In the reirradiation setting, dose prescription must balance tumor control with the cumulative tolerance of surrounding neural structures, with SRS primarily reserved for small recurrences, including WHO grade 2 and 3 disease after adjuvant RT [20], [21]. In this context, the STORM multicenter analysis specifically evaluated salvage SRS for recurrent WHO grade 2 and 3 meningiomas, including a reirradiated subgroup. Single-fraction treatments were delivered to a median marginal dose of 16 Gy (range 14.8–18 Gy), supporting the feasibility of prescription doses in the 14–18 Gy range in carefully selected patients, while acknowledging the increased risk of RN in previously irradiated volumes [24]. For lesions unsuitable for single-fraction treatment, hypofractionated stereotactic regimens of 25–30 Gy in 5 fractions are commonly used, supported by favorable safety profiles in several reirradiation cohorts [14], [18], [22]. When larger volumes or skull base locations require lower doses per fraction, conventional schedules of 50–54 Gy in 1.8–2 Gy fractions have been employed, particularly in proton or high-precision photon treatments, with acceptable side effects and meaningful local control [5], [14], [25]. Overall, these dose ranges reflect the need to tailor treatment to lesion size, location, prior exposure, and expected tolerance of the organs at risk. A recent systematic review and meta-analysis on meningioma reirradiation estimated the risk of symptomatic RN to be 8%, with risks of acute and late side effects of any grade of 22 and 16%, respectively [16]. The risk of high-grade side effects was low.

In summary, reirradiation of intracranial meningiomas is a feasible strategy that provides meaningful disease control in selected patients, particularly when high-precision techniques are used, and treatment intervals allow partial repair of normal tissue. In the absence of urgently needed prospective trials, the most robust evidence indicates that SRS and FSRT demonstrate favorable safety and efficacy across many settings, and that particle therapy may provide dosimetric advantages for specific scenarios, such as complex skull base recurrences. The risk of side effects is most strongly influenced by target volume, cumulative dose, and lesion proximity to critical structures, whereas tumor grade, performance status, and the interval since prior RT are among the factors influencing treatment outcomes. The role of molecular risk stratification to refine management is an ongoing area of investigation [26], [27]. The integration of these data into clinical decision-making supports a personalized approach, balancing the competing goals of tumor control and preservation of neurological function.

## 3 Ependymoma

Ependymomas are rare CNS tumors of neuroectodermal origin that occur across all age groups, although a substantial proportion are diagnosed in young children. In current clinical practice, maximal safe surgical removal, followed by adjuvant RT to the tumor bed or craniospinal irradiation, depending on the extent of resection, presence of metastases, and tumor grade, constitutes the standard therapeutic approach. Adjuvant RT in children is employed for all intracranial grade 2 and 3 tumors, whereas in adults, postoperative RT is utilized in grade 3 tumors regardless of the extent of resection and in incompletely resected grade 2 disease. Despite optimal local treatment, recurrence develops in roughly one-third to one-half of patients [28]. At relapse, management typically relies on further local control through repeat surgery when feasible and consideration of reirradiation, as systemic therapies have shown limited efficacy.

Growing evidence indicates that reirradiation can provide meaningful benefit in both adult and pediatric populations, whether delivered as conventionally fractionated radiotherapy, stereotactic irradiation, or proton therapy, although the duration of disease control may vary (Table 2). In adults, it is increasingly considered for recurrent intracranial ependymoma when surgery alone is insufficient or not feasible, but the evidence remains limited and largely retrospective, making individualized, multidisciplinary decision-making essential [29]. When approaching a patient with recurrent intracranial ependymoma, the initial question is always whether a second surgery can achieve a safe gross total resection. Multiple series have demonstrated that the extent of resection remains one of the strongest determinants of subsequent progression-free and overall survival, and, therefore, attempts at maximal safe resection should be pursued when anatomically and clinically feasible before considering reirradiation [30].

**Table 2:** Selected studies on reirradiation of ependymomas.

Author/Year	Study design	N patients	WHO Tumor grade	First Course RT technique(s)	First Course RT dose (Gy)/fractions (fx)	Re-RT technique(s)	Re-RT dose (Gy)/fractions (fx)	Outcomes (PFS/OS)	RN
Eaton et al., 2015	Retrospective	20 pediatric (33 lesions)	1–3	EBRT, PT	Median dose of 55.8 Gy (range 52.2–59.4)	Proton therapy	Median dose 50.4 GyRBE (35–55.8)	mPFS: 19.1 mo PFS 3-yr: 28.1% OS 3-yr: 78.6%	Grade 2 radiation-associated treatment change: 21.4%; RN not separately reported.
Murai et al., 2016	Retrospective	8 (38 lesions)	1–3	EBRT	RT dose range 44–60 Gy	SRS, FSRT	Median: 15.3 Gy (10.4–23)/1 fx 20 Gy/2 fx 22.6 Gy (18–35)/3 fx 23.4 Gy (18–35)/5 fx	mPFS: 8 mo PFS 3-yr: 21% mOS: 24 mo	No toxicity ≥ grade 2
Régnier et al., 2019	Retrospective	31 (pediatric)	2–3	EBRT	57.6 Gy (range, 48.6 to 61) bifractionated protocol with 60 Gy/60 fx (3 patients)	SRS, FSRT, EBRT	18 Gy/1 fx 24–51 Gy /3–10 fx 27–59.4 Gy /15–30 fx	mPFS 31 months (range, 2 to 63) mOS 34 months (range, 3 to 63).	1 patient required corticosteroids
Gupta et al., 2020	Retrospective	55 (pediatric)	1–3	EBRT	Median dose EQD2 54.8 Gy (44.3–60)	EBRT	54 Gy (40-60)/30 fx	PFS 3-yr: 40% OS 3-yr: 51%	Symptomatic: 5.5% (3 patients)
Yoo et al., 2024	Retrospective	34 (60/75 lesions were recurrent, pediatric and adult, outcomes for the reirradiated subgroup were not separately reported)	2–3	NR	NR	SRS	16.6 Gy/1 fx median single-fraction equivalent dose	PFS 5-yr: 68.5% OS 5-yr: 73.6%	NR

**EBRT**, external beam radiotherapy; **EQD2**, equivalent dose in 2-Gy fractions; **FSRT**, fractionated stereotactic radiation therapy; **fx**, fractions; **mo**, months; **mOS**, median overall survival; **mPFS**, median progression-free survival; **NR**, not reported; **OS**, overall survival; **PFS**, progression-free survival; **PT**, proton therapy; **RBE**, relative biological effectiveness; ***Re*-RT**, reirradiation; **RN**, radiation necrosis; **RT**, radiation therapy; **SRS**, stereotactic radiosurgery; **yr**, year.

If complete re-resection is not achievable, the patient prefers a non-invasive treatment, or recurrence is multifocal or disseminated, reirradiation becomes the principal local salvage modality. Contemporary practice has evolved to employ either conventionally fractionated focal reirradiation, SRS, FSRT, or proton therapy, with the choice guided by prior dose distribution, target size and location, time since initial RT, and proximity to critical structures [1], [31].

Conventionally fractionated reirradiation has been shown to be feasible in selected patients, allowing delivery of cumulative therapeutic doses with acceptable side effects [32], [33]. Hypofractionated approaches with FSRT, such as 21 Gy in 3 fractions or 25 Gy in 5 fractions, may provide satisfactory short-term local control with limited severe adverse effects, whereas single-fraction SRS must be tailored carefully because of prior exposure of the brainstem or optic apparatus, and lesion size can significantly limit dose escalation [34], [35].

The risk of adverse events after reirradiation is significant but appears acceptable in carefully selected adult patients treated with modern techniques and meticulous planning. Retrospective pediatric and mixed-age series have reported RN rates that are generally low for focal reirradiation when cumulative high-dose volumes are restricted, although brainstem and cranial nerve toxicities can be severe with salvage SRS after prior high-dose exposure. Lesion location relative to eloquent structures must govern feasibility and dose prescription [30], [34], [36]. When comparing techniques, proton data are more mature in pediatric cohorts, and the adult intracranial ependymoma experience remains limited. Therefore, technique selection should be based on dosimetry, accessibility, cost, and the specific pattern of prior radiation exposure [1], [36].

Given the heterogeneity and retrospective nature of published reports, the available adult-focused evidence supports the following pragmatic clinical approach: pursue maximal safe re-resection when feasible, evaluate prior dose distributions and organ at risk exposure, consider proton therapy when dosimetric advantages translate into clinically meaningful sparing of critical structures, and employ stereotactic techniques for small targets while using conventionally fractionated focal reirradiation in cases where tumor size or proximity to critical structures dictates fractionation to limit side effects [1], [32].

In summary, reirradiation for intracranial ependymoma is a viable salvage strategy when tailored to the clinical scenario, with technique and dose selected based on prior therapy, target characteristics, and the need to preserve critical structures. Involvement of a multidisciplinary team is strongly advised to discuss the balance between disease control and the risk of radiation-induced injury. Finally, whenever possible, patient enrollment in prospective registries or collaborative protocols is recommended to strengthen the evidence for reirradiation in this rare patient population [1].

## 4 Brain metastases

Type 1 reirradiation for brain metastases has emerged as an increasingly relevant therapeutic strategy in contemporary neuro-oncology, largely driven by improvements in systemic therapies and intracranial surveillance, which have extended survival and consequently increased the likelihood of intracranial recurrence after initial RT [37], [38]. In this evolving clinical context, management of recurrence requires a nuanced understanding of tumor biology, treatment history, and the dynamic interplay between local and systemic approaches. Moreover, the accurate differentiation between RN and true local tumor progression warrants careful assessment of imaging findings. The use of advanced imaging modalities, including perfusion-weighted MRI, contrast clearance analysis (CCA), MR spectroscopy, amino-acid positron emission tomography (PET), and advanced diffusion techniques, may improve diagnostic confidence [39], [40].

Once recurrence is strongly suspected based on clinical and imaging findings, the clinical approach to recurrent brain metastases depends on lesion size, location, prior treatment modality, time to recurrence, status of extracranial disease, availability of systemic treatment options, and patient performance status [37], [41]. Recurrent lesions within prior high-dose regions often demonstrate more resistant biological behavior and require individualized decision-making. Surgery is appropriate for large or symptomatic accessible lesions, and retrospective evidence suggests improved outcomes when surgical resection is followed by adjuvant radiation [42]. Wilcox and colleagues reported meaningful improvements in local control with this combined approach, supporting its use in selected cases [43].

As systemic therapies become more effective and capable of eliciting intracranial responses, optimal integration of CNS-active agents is increasingly integral to the management of recurrent disease. Importantly, intracranial progression may exhibit molecular divergence from extracranial lesions, driven by selective pressure of the CNS microenvironment, blood-brain barrier dynamics, and prior RT [37]. Research by Brastianos and colleagues demonstrated frequent genomic divergence between primary tumors, extracranial metastases, and brain metastases, identifying distinct actionable alterations in intracranial lesions that could inform subsequent lines of therapy [44]. Similar patterns of molecular evolution have been observed in lung cancer, where intracranial progression may develop new resistance mutations relevant to targeted agents, reinforcing the value of tissue sampling or liquid biopsy when clinically appropriate [45]. Within this framework, reirradiation becomes one component of a broader multimodal strategy in which CNS-active systemic therapies play an increasingly decisive role.

Dose and fractionation strategies for reirradiation reflect the need to balance tumor control with preservation of normal brain function (Table 3). Type 1 reirradiation using single-fraction SRS typically employs doses of 15–18 Gy for small recurrences, informed by both clinical experience and the dose limits established in RTOG 90–05, which identified safe upper boundaries for lesions of different sizes after prior whole-brain RT [46]. For larger lesions or those in proximity to eloquent structures, hypofractionated regimens such as 21–24 Gy in 3 fractions or 25–30 Gy in 5 fractions are preferred, with multiple retrospective series supporting improved safety profiles in previously irradiated tissues [47], [48]. In patients with recurrence after previous brain RT (whole-brain RT, partial-brain RT), salvage SRS with a median dose of 21 Gy (range 12–24) has demonstrated durable control (46.8% at 3 years) and survival (median overall survival 11.7 months) with modest side effects [49]. Whole-brain reirradiation is reserved for highly selected cases with widespread intracranial progression, and contemporary practice favors reduced doses, such as 20 Gy in 10 fractions, to mitigate severe neurocognitive decline [50]. Repeat SRS for new metastases without geometric overlap (type 2 reirradiation or repeat organ irradiation, depending on the location of the new brain metastasis and prior treatment fields) is commonly used to address progressive CNS disease [51], [52].

**Table 3:** Selected studies on reirradiation of brain metastases.

Author/Year	Study design	N patients	First Course RT technique(s)	First Course RT dose (Gy)/fractions (fx)	Re-RT technique(s)	Re-RT dose (Gy)/fractions (fx)	Outcomes (PFS/LC/LR/OS)	RN
Wilcox et al., 2021	Retrospective	135 (155 lesions, A: 116 SR B: 39 SR + PSRT)	SRS ± WBRT, Surgery +SRS ± WBRT	NR	FSRT/SRS/WBRT	NR	A: LR 1-yr: 43.9% B: LR 1-yr: 28.8%	Symptomatic RN: A: 0.9% B: 5.1%
Minniti et al., 2016	Retrospective	43 (47 lesions)	SRS	NR	FSRT	21 Gy/3 fx, 24 Gy/3 fx	LC 1-yr: 70% LC 2-yr: 60% OS 1-yr: 37% OS 2-yr: 20%	19% (9 lesions)
Shaw et al., 2000	Prospective dose-escalation trial	156 64% BM 36% PBT	NR	Median prior dose 30 Gy	SRS	≤ 20 mm (largest diameter): up to 24 Gy 21–30 mm: 18 Gy 31–40 mm: 15 Gy	LR in 48% overall, higher risk of LR in primary brain tumors vs. metastases	6 mo: 5% 12 mo: 8% 18 mo: 9% 24 mo: 11%
Yan et al., 2023	Retrospective	91 (120 lesions)	SRS, FSRT	16–20 Gy/1 fx 24–35 Gy/3–5 fx	FSRT	20–35 Gy/5 fx 20–24 Gy/3 fx	LR 6 mo: 18.9% LR 12 mo: 27.7%	Radiographic RN: 6 mo: 13% 12 mo: 15.6% Symptomatic RN: 6 mo: 6.1% 12 mo: 7.0%

**BM**, brain metastases; **FSRT**, fractionated stereotactic radiation therapy; **fx**, fractions; **LC**, local control; **LR**, local recurrence; **mo**, months; **NR**, not reported; **OS**, overall survival; **PBT**, primary brain tumor; **PSRT**, post-salvage radiation therapy; **RN**, radiation necrosis; **SR**, salvage resection; **SRS**, stereotactic radiosurgery; **WBRT**, whole-brain radiation therapy; **yr**, year.

Therefore, reirradiation for brain metastases represents a viable and increasingly sophisticated strategy that integrates radiobiological understanding, evolving systemic therapies, and improved imaging. Contemporary practice emphasizes individualized therapy that balances local control with neurological preservation, supported by a growing evidence base and refined by multidisciplinary assessment.

## 5 PitNETs

In 2022, the WHO published the 5^th^ edition of the Classification of Endocrine and Neuroendocrine Tumors, introducing substantial changes in the definition and categorization of pituitary tumors [53]. In this edition, pituitary adenomas have been officially redefined as PitNETs, acknowledging that, although these lesions are generally slow-growing and often clinically indolent, they share biological features with neuroendocrine tumors arising in other organs. The new classification emphasizes the cell lineage of origin, determined by immunohistochemistry for the key transcription factors: PIT1, TPIT, SF1, GATA3, and ERα. Based on this approach, PitNETs are categorized into four groups: PIT1 lineage, TPIT lineage, SF1 lineage, and not otherwise specified (null cell tumors and unclassifiable plurihormonal PitNETs). Epidemiologically, PitNETs occur with an estimated annual incidence of approximately 5 cases per 100,000 individuals, and they account for 17.4% of all primary brain and other CNS tumors, with a greater incidence after 40 years of age [54].

From a therapeutic perspective, RT remains a well-established primary option for both functioning (FAs) and non-functioning (NFAs) adenomas, used for long-term tumor control and achievement of biochemical remission. Conventional fractionated radiation therapy typically delivers 45–50.4 Gy in daily 1.8 Gy fractions over 5–6 weeks. For non-functioning tumors, 14–16 Gy is delivered for single-fraction SRS, and for FSRT, 21 Gy in 3 fractions and 25 Gy in 5 fractions can be utilized [55], [56], [57], [58]. For FAs, single-fraction SRS doses of 16–25 Gy have been used, with some selected series reporting single-fraction doses of up to 35 Gy, while 21–24 Gy in 3 fractions and 25–32 Gy in 5 fractions have been reported for FSRT [59], [60], [61], [62], [63], [64].

Despite their generally favorable long-term outcomes, PitNETs carry a non-negligible risk of recurrence after surgery. For NFAs, after gross total resection, recurrence rates range between 7 and 12% at 10 years [65]. A meta-analysis of >900 patients further confirmed a pooled recurrence rate of 12% after complete resection [66]. When considering the entire cohort, including incomplete resections and residual disease, cumulative regrowth rates were 25%, 43%, and 61% at 5, 10, and 15 years, respectively. Among patients with surgical residuals, the regrowth rate in a population of 100 patients was 47% [67], and the regrowth risk seems to be 3.7 times higher when the remnants are located outside the sellar region [68]. RT, whether delivered as SRS/SRT or fractionated techniques, has been shown to provide durable tumor control in both non-functioning and functioning pituitary adenomas, with long-term response reported in the majority of patients across large series and meta-analyses [55], [59].

Within this context, reirradiation has emerged as an increasingly relevant treatment option for patients with progressive, non-operable disease refractory to medical therapy or patients not eligible for further surgery (Table 4). Minniti et al. retrospectively evaluated 21 patients who underwent reirradiation with 36 Gy in 2 Gy fractions or 37.5 Gy in 2.5 Gy fractions combined with Temozolomide (TMZ), achieving 2-year and 4-year local control rates of 73% and 65%, respectively [69]. Comparable local control rates of 80% and 58% at 2 and 5 years, respectively, were reported for 15 patients by Verma et al., using median doses of 45 Gy (27.9–54 Gy) for fractionated RT and 18 Gy for SRS [70]. Nevertheless, just a small portion of patients ultimately undergo reirradiation, reflecting the highly selective nature of this approach. This selectivity is largely driven by the typical sellar and parasellar location of PitNETs, where recurrent or residual disease may lie close to critical structures such as the optic nerves, optic chiasm, and temporal lobes, making cumulative dose assessment essential. The clinical relevance of this proximity is illustrated by the retrospective series on reirradiation from Verma et al., in which RION occurred in 13.3% of patients (2/15) (9% at 5 years), and temporal lobe necrosis (TLN) in 13.3% of cases (2/15) (10% at 2 years, 28% at 5 years) though these estimates derive from a small cohort and should be interpreted with caution [70].

**Table 4:** Selected studies on reirradiation of PitNETs.

Author/Year	Study design	N patients	Tumor subtype	First Course RT technique(s)	First Course RT dose (Gy)/fractions (fx)	Re-RT technique(s)	Re-RT dose (Gy)/fractions (fx)	Outcomes (PFS/LC/OS)	RN
Verma et al., 2014	Retrospective	15	NFA 6, CD 5, Acro 3, PRL 1	EBRT/SRS	Median: 49.5 Gy EBRT, 15–25 Gy SRS.	EBRT/SRS/FSRT/brachytherapy	Median: 45 Gy EBRT (27.9–54), 18 Gy SRS	LC 2-yr: 80% LC 5-yr: 58%	13.3% (2 patients, treated with SRS)
Minniti et al., 2020	Retrospective	21	APT 17; PC 4	EBRT/SRS	45–50.4 Gy/25–28 fx	FSRT	36 Gy/18 fx, 37.5 Gy/15 fx	LC 2-yr: 73% LC 4-yr: 65% OS 2-yr: 82% OS 4-yr: 66% (Patients received TMZ)	Grade 3 radiation-related toxicity: 14% (3/21); RN not separately reported
Li et al., 2021	Retrospective	12	NFA	SRS	Median margin dose 10 Gy (range, 9.0–15.5 Gy)	SRS	Median margin dose 12 Gy (10–14 Gy)	LC 1-yr: 100% LC 5-yr: 90% LC 10-yr: 68%	NR
Lindberg et al., 2024	Retrospective	23	Mixed (NFA 61%, ACTH 17%, GH 8.7%, PRL 13%)	SRS	NR	SRS	Max dose: 44 Gy (40–50 Gy), Marginal prescription dose: 22 Gy (20–25 Gy)	PFS 5-yr: 57% Median TTP: 2.6 yr	NR

**Acro**, acromegaly; **ACTH**, adrenocorticotropic hormone; **APT**, aggressive pituitary tumor; **CD**, Cushing Disease; **EBRT**, external beam radiotherapy**; FSRT**, fractionated stereotactic radiation therapy; **fx**, fractions; **GH**, growth hormone; **LC**, local control; **mo**, months; **mOS**, median overall survival; **NFA**, non-functioning pituitary adenoma; **NR**, not reported; **OS**, overall survival; **PC**, pituitary carcinoma; **PFS**, progression-free survival; **PitNETs**, pituitary neuroendocrine tumors; **PRL**, prolactinoma; ***Re*-RT**, reirradiation; **RN**, radiation necrosis; **RT**, radiation therapy; **SRS**, stereotactic radiosurgery; **TMZ**, temozolomide; **TTP**, time to progression; **yr**, year.

Among late effects, endocrine dysfunction remains the most frequent and is often unavoidable, whereas visual and temporal lobe injuries, although less common, have a significant impact on quality of life. The frequency of growth hormone (GH) deficiency reaches 50–100% within 3–5 years after cranial irradiation with high radiation doses (30–50 Gy). Thyroid-stimulating hormone (TSH) and adrenocorticotropic hormone (ACTH) deficits are generally less frequent, with cumulative incidences increasing with higher radiation doses and longer follow-up, particularly after doses ≥50 Gy. Gonadotrophin deficiency is uncommon and typically represents a late complication, usually reported after cumulative doses of ≥30 Gy. Following stereotactic radiotherapy, hypopituitarism is predominantly a long-term effect, with reported incidences broadly comparable to those observed after conventionally fractionated irradiation [71], [72]. Thus, reirradiation should always account for this aspect when employed. Although comparative evidence between modalities is limited, stereotactic techniques and fractionated radiotherapy offer improved conformality and limited treatment-related toxicities. Hormonal deficiencies are often delayed and may continue for up to 10 years after treatment [55].

More recently, a case report described durable disease control in an ACTH-secreting pituitary carcinoma treated with image-guided IMRT (56 Gy in 28 fractions). Reirradiation combined with concurrent TMZ (75 mg/m^2^ for 42 days) resulted in sustained biochemical remission and radiological response for nearly 2 years, with an overall survival exceeding 46 months [73]. While the evidence is limited, this observation highlights the potential feasibility of multimodality approaches integrating reirradiation and TMZ in highly aggressive, refractory cases.

Proton therapy offers dosimetric advantages in the reirradiation of PitNETs, although clinical evidence in the reirradiation setting remains scarce [74]. However, due to the lack of prospective trials and long-term follow-up data, and uncertainty about its relative biological effectiveness, its current use in PitNET reirradiation should be considered on a case-by-case basis in experienced centers and preferably within prospective registries.

In summary, reirradiation represents a feasible and potentially effective salvage option for recurrent PitNETs when surgery and medical therapy fail. Stereotactic techniques may offer a favorable balance between treatment effectiveness and side effects, provided that cumulative doses to the optic pathways and hypothalamic-pituitary structures are rigorously respected. Prospective studies are needed to refine optimal dose and fractionation strategies and to better characterize long-term side effects, reporting data in accordance with the ESTRO-EORTC consensus recommendations to facilitate consistency and comparability across studies [3].

## 6 Radiation necrosis

### 6.1 Imaging and diagnosis

RN is a delayed and potentially debilitating complication of high-dose CNS irradiation, arising from vascular injury, blood-brain barrier disruption, and progressive white matter damage that culminates in demyelination and necrosis [1], [75]. Clinically, RN occurs 6–24 months after RT, although later presentations have been reported [76], and it often mimics tumor recurrence, presenting with seizures, headache, focal neurological deficits, or cognitive decline, typically [77]. Therefore, distinguishing RN from actual progression is one of the greatest unmet needs in neuro-oncology.

The incidence of RN depends on tumor histology, total delivered dose, irradiated volume, fractionation, concurrent therapies, proximity to critical structures, including deep white matter tracts and the ventricular system, and other patient-specific factors [78], [79]. These findings are supported by other studies [80]. Recent mechanistic modeling studies further support a multifactorial pathogenesis. In addition, vascular heterogeneity might contribute to the development and RN expansion patterns, suggesting potential applications for treatment planning and adaptive follow-up strategies [81]. Overall, reported incidence rates vary considerably, ranging from approximately 4% to 30%, while symptomatic RN is generally reported in up to 10% of patients [77].

After conventional fractionated RT for brain tumors, symptomatic RN develops in approximately 5–10% of patients and has been reported at cumulative doses around 60–90 Gy EQD2 (α/β = 2) [82]. In single-fraction SRS, the risk of radiation-induced complications rises steeply when the volume of brain receiving 12 Gy (V12) exceeds 5–10 ml [83]. The risk increases substantially in the reirradiation setting, where cumulative dose becomes a critical determinant. An updated review of the glioblastoma reirradiation literature confirmed symptomatic RN rates between 0 and 24% at 1 year [82]. The risk generally remains below 10% when the cumulative brain EQD2 (α/β = 2 Gy) is kept under 100–110 Gy, but it rises to approximately 25% beyond 130 Gy [1]. In addition, there is a notable volume effect that must be considered.

For single-fraction SRS, dose-volume metrics are among the strongest predictors, although the volume definitions used across studies are not always uniform. A retrospective series on 310 cerebral metastases reported an increased RN risk for V10 Gy and V12 Gy exceeding 12.6 cm^3^ and 10.9 cm^3^, respectively [83]. In this study, dose-volume metrics were calculated from the volume of brain receiving each threshold dose. Milano et al. reported that symptomatic RN (using brain plus gross tumor volume [GTV]) occurred in approximately 10%, 15%, and 20% of cases when the V12 was 5 cm^3^, 10 cm^3^, and > 15 cm^3^, respectively [84]. Consistent with these findings, the recent EANO consensus identified a normal-brain V12 of approximately 10 cm^3^, defined by subtracting the GTV and non-brain tissue, as being associated with a 5–10% risk of symptomatic RN [77].

For FSRT, recent data provide additional dose-volume thresholds. In a large 3-fraction cohort, when considering brain plus GTV, V20 and V23 emerged as the most robust predictors of RN, with cut-offs of 4 cm^3^, 10 cm^3^, and 20 cm^3^ associated with <5%, <7.5%, and <10% risk of any RN, and V23 < 15 cm^3^ correlating with <5% risk of grade ≥ 3 symptomatic RN [85]. The EANO consensus also reports that a combined brain-plus-target V20 Gy after three fractions or V24 Gy after five fractions below 20 cm^3^ has been associated with a <10% risk of any necrosis or edema and a < 4% risk of RN requiring surgical resection [77]. In patients treated with 5-fraction FSRT, local control was highest with doses ≥30 Gy delivered in 5 fractions, with symptomatic RN observed in approximately 9–10%, supporting the safety of this regimen when constraints are respected [86]. Summation of composite doses across all prior RT courses is essential before considering any additional irradiation.

Additional risk factors include larger treatment volumes, shorter intervals between radiation courses, and concurrent systemic therapies. In this regard, immune checkpoint inhibitors (ICIs) may potentiate inflammatory or vascular injury and thereby increase RN risk [87], although the recent ESMO-ESTRO consensus indicates that the overall side-effect profile of combining RT with ICIs is generally favorable, particularly for PD-(L)1 inhibitors. On the contrary, caution is recommended when RT is administered with VEGF(R) inhibitors or multitargeted tyrosine kinase inhibitors (TKIs), given the limited high-quality side effects data and the potential for amplified treatment-related adverse effects [88]. Therefore, while concurrent bevacizumab may be considered in selected high-risk reirradiation cases — especially for large treatment volumes or patients with significant edema — its use should be individualized and carefully monitored.

Taken together, these clinical and systemic factors must be balanced against the radiobiological constraints of normal tissue tolerance. Radiobiological evidence supports partial recovery of CNS tolerance, enabling cumulative EQD2 (α/β = 2 Gy) limits of ∼100–110 Gy in selected cases [89]. Proton therapy may offer advantages in dose sparing, but its role must be further defined due to uncertainties about its relative biological effectiveness and LET (linear energy transfer)-related effects near the distal edge, particularly in the subventricular zone [90], [91]. Photon-based stereotactic approaches (SRS, FSRT), therefore, remain the most used modalities for reirradiation.

Differentiating RN from tumor recurrence remains notoriously challenging due to overlapping radiological features, such as contrast enhancement, perilesional edema, mass effect, and progressive enlargement in or near the initial treatment field, as well as clinical symptoms [92], [93]. RN and viable tumor may also coexist within the same lesion, further complicating both diagnosis and management [76]. In this context, it is also important to discriminate pseudoprogression, a transient treatment-related effect characterized by new or increased enhancement that subsequently stabilizes or resolves without any change in therapy [94], [95].

Pathological confirmation after biopsy or surgical resection remains the gold standard for diagnosing RN [[77], [78]]. However, its diagnostic value may be limited by the lack of universally standardized histopathological criteria and, in irradiated gliomas, by the difficulty of identifying viable tumor cells because of overlapping cytological features between neoplastic cells and reactive glia [76]. In clinical practice, tissue confirmation is not always feasible or indicated, and diagnosis commonly relies on multidisciplinary integration of imaging features, clinical evolution, and prior RT dose distribution. Therefore, imaging findings should be interpreted as probabilistic or suggestive of necrosis, rather than definitive. Because conventional imaging criteria alone lack sufficient specificity in distinguishing RN from progression, advanced imaging techniques play a central role. Perfusion MRI, diffusion sequences, and MRI spectroscopy can help guide the RN diagnosis [96]. Relative cerebral blood volume (rCBV) derived from DSC (dynamic susceptibility contrast)-MRI is among the most robust parameters for differentiating between these two entities [97], [98]. Recurrent tumors typically exhibit elevated rCBV due to neovascular proliferation, whereas RN is associated with reduced rCBV reflecting impaired microvascular perfusion [92], [99]. Other studies even suggest that rCBV may outperform amino-acid PET in certain contexts, although prospective validation is still needed. Amino-acid PET imaging (18F-FET, 11C-MET) remains highly valuable for obtaining metabolic information, potentially improving discrimination between tumor and treatment-related changes. It may also offer practical workflow advantages, particularly when integrated with hybrid PET/MRI platforms, which allow simultaneous acquisition of metabolic and MRI parameters within a single session [100]. The main limitations of PET imaging are cost and limited on-demand availability of amino acid tracers. In addition to perfusion and diffusion imaging, CCA, also referred to as Treatment Response Assessment Maps (TRAMs), has emerged as a promising tool for differentiating RN from tumor progression. Recent studies have shown that CCA achieves high diagnostic accuracy, with pooled sensitivity and specificity around 90% in distinguishing posttreatment changes from true progression, outperforming conventional MRI and adding valuable information in ambiguous cases [101].

Overall, the diagnosis of RN requires a thorough understanding of its spatiotemporal occurrence and careful consideration of available imaging and previous RT courses. When observation is chosen, MRI follow-up at 6- to 12-week intervals is reasonable, with the timing tailored to symptom severity and the clinical course. Amino-acid PET may be used to increase diagnostic confidence at baseline, during follow-up, or when assessing response to RN-directed treatments.

### 6.2 Clinical management of RN

The recent EANO consensus recommends that radiological findings suggestive of RN alone should not prompt pharmacological treatment in asymptomatic patients, and treatment should be considered when symptoms persist for more than seven days or progressively increase in severity [77]. When therapeutic management is required, it typically follows a stepwise strategy. The International Stereotactic Radiosurgery Society (ISRS) summarized the available evidence and proposed a structured clinical algorithm for the management of symptomatic and steroid-refractory RN [102].

Corticosteroids, such as dexamethasone, have long been the first-line therapy, offering rapid symptom relief by reducing vasogenic edema. The EANO consensus on RN recommends using the lowest dose that provides meaningful clinical benefit, guided primarily by the patient's symptom burden [77]. A reasonable starting dose is 1.5–4 mg of dexamethasone once daily in the morning for mild-to-moderate symptoms and up to 8 mg daily for severe symptoms, initially administered for 3–5 days. The dose may subsequently be reduced by approximately 2 mg every 3–5 days according to the clinical response. A maintenance dose of approximately 1.5–2 mg may be continued until the first follow-up imaging and subsequently reduced or discontinued in the presence of combined clinical and radiological benefit [77].

For steroid-refractory or steroid-dependent cases, bevacizumab plays a central role in the RN management. Randomized controlled trials, including the pivotal study by Levin et al., showed both radiographic and symptomatic improvements [103]. The efficacy of bevacizumab is further supported by the ISRS systematic review, which reported clinical improvement or stability rates of 86% and radiographic improvement or stability rates of 93–94% across thirteen studies, leading to a strong recommendation for its use in steroid-refractory RN [102]. The EANO consensus proposes considering a switch after approximately four weeks when the clinical benefit from corticosteroids remains insufficient, particularly in steroid-dependent patients requiring around 8 mg/day of dexamethasone or an equivalent dose [77]. No optimal bevacizumab dose, schedule, or duration has been established, and clinical and radiological responses have been reported with both conventional and low-dose regimens, generally administered for less than three months. Potential adverse effects, particularly thromboembolic events, hypertension, bleeding, and impaired wound healing, should be considered. Bevacizumab should not be initiated when surgical resection is considered the preferred or likely subsequent treatment option [77].

There is emerging clinical evidence – derived mainly from small case series – that suggests boswellia serrata (BS) and its extracts may be beneficial in managing cerebral radiation-induced edema or RN [104]. In one pilot randomized-controlled trial in irradiated brain tumor patients, patients received either BS (4200 mg/day) or a placebo during RT. A reduction of cerebral edema >75% (from baseline) was observed in 60% of BS patients vs. 26% in placebo on T2-weighted MRI [105]. BS also appears to be well tolerated, with adverse effects largely limited to mild gastrointestinal symptoms and no significant signals of side effects to date [106]. Importantly, this approach may offer a steroid-sparing effect, which is particularly relevant given the well-known risks of chronic corticosteroid therapy.

Such steroid-sparing strategies are especially attractive in patients receiving modern treatments, including immunotherapy, where minimizing long-term immunosuppression is desirable. Nevertheless, the current evidence remains very limited and insufficient to support definitive clinical recommendations, underscoring the need for robust, prospective, and adequately powered trials.

Surgical resection should be considered whenever safely feasible, particularly for accessible lesions associated with rapid neurological deterioration, substantial mass effect, diagnostic uncertainty, failure of medical therapy, or an anticipated need for prolonged treatment. Biopsy may be considered when diagnostic confirmation is required, but complete resection is not appropriate, although sampling bias and the coexistence of RN and viable tumor remain important limitations [77].

Minimally invasive techniques such as laser interstitial thermal therapy (LITT) are increasingly adopted, supported by new data and emerging evidence suggesting comparable outcomes to bevacizumab [107], although randomized comparative studies are lacking. The role of hyperbaric oxygen therapy (HBOT) in RN management has also been explored in small series. Reported outcomes show HBOT as a feasible and effective therapeutic option in steroid-refractory RN, associated with favorable outcomes and a low rate of side effects [108]. However, its role remains investigational.

In summary, RN represents a clinically significant side effect in the intracranial reirradiation setting, requiring multiparametric imaging and multidisciplinary evaluation. Asymptomatic radiological abnormalities can generally be managed with close observation, whereas symptomatic RN requires individualized treatment. Early recognition, adherence to proposed dose-volume constraints, and timely initiation of medical or surgical interventions are essential to preserve neurological function and optimize quality of life.

## 7 Conclusion

This pragmatic review by the ESTRO CNS Focus Group reports on the role of reirradiation in the management of various brain tumors, including meningioma, ependymoma, brain metastases, and PitNETs. With growing interest in reirradiation and the need to evaluate its potential efficacy and safety across tumor entities, future work in the field must be accompanied by thoroughly designed, well-structured prospective studies. Strict reporting in accordance with published recommendations from the ESTRO-EORTC and Reirradiation Collaborative Group (ReCOG) is essential to ensure that evidence from future studies is of the highest quality.

## CRediT authorship contribution statement

**Raffaella De Pietro:** Methodology, Investigation, Writing – original draft, Writing – review & editing, Visualization. **Elena Clerici:** Methodology, Investigation, Writing – original draft, Writing – review & editing, Visualization. **Maciej Harat:** Writing – review & editing. **Karthica Indramohan:** Writing – review & editing. **Tomas Kazda:** Writing – review & editing. **Slavka Lukacova:** Writing – review & editing. **Andrada Turcas:** Writing – review & editing. **Giuseppe Minniti:** Conceptualization, Methodology, Writing – review & editing, Writing – original draft, Supervision. **Maximilian Niyazi:** Conceptualization, Methodology, Writing – review & editing, Writing – original draft, Supervision. **Felix Ehret:** Conceptualization, Methodology, Writing – review & editing, Writing – original draft, Supervision.

## Declaration of competing interest

The authors declare the following financial interests/personal relationships which may be considered as potential competing interests: Maciej Harat has received honoraria and travel support from Servier and Brainlab, and acknowledges research funding from the Polish Agency of Medicine Research, all unrelated to this work. Slavka Lukacova has received honoraria from Servier, unrelated to the submitted work. Giuseppe Minniti has received honoraria for lectures, consultation, or advisory board participation from Brainlab, Accuray, Servier, AstraZeneca, Pfizer, and Novocure. Maximilian Niyazi has received honoraria from Elekta, Brainlab, and AstraZeneca, and acknowledges research funding and contractual research support from TheraPanacea, Elekta, and Carl Zeiss Meditec (including advisory board activities), all unrelated to the submitted work. Felix Ehret has received honoraria and travel support from ZAP Surgical Systems, Inc., and Accuray, Inc., and acknowledges research funding from the German Cancer Aid and Accuray, Inc., all unrelated to the submitted work.
